# Cerebellar gray matter atrophy and altered structural covariance networks are associated with cognitive impairment in type 2 diabetes mellitus

**DOI:** 10.3389/fneur.2025.1626518

**Published:** 2025-10-09

**Authors:** Meixia Long, Jinyun Xue, Dingwen Hu, Hui Chu, Shijun Qiu

**Affiliations:** ^1^The First Clinical Medical College, Guangzhou University of Chinese Medicine, Guangzhou, China; ^2^Department of Radiology, The First Affiliated Hospital of Guangzhou University of Chinese Medicine, Guangzhou, China; ^3^State Key Laboratory of Traditional Chinese Medicine Syndrome, Guangzhou, China

**Keywords:** type 2 diabetes mellitus, cerebellar gray matter, caudate nucleus, voxel-based morphometry, structural covariance network, cognitive impairment, default mode network

## Abstract

**Background:**

The prevalence of type 2 diabetes mellitus (T2DM) is steadily increasing, with central nervous system complications commonly manifesting as mild cognitive impairment and dementia. However, the neuropathophysiological mechanisms underlying T2DM-related cognitive dysfunction remain poorly understood.

**Method:**

This study used voxel-based morphometry (VBM) and seed-to-voxel structural covariance network (SCN) analyses to investigate alterations in cerebellar gray matter volume (GMV) and SCNs in T2DM, as well as their associations with cognitive performance. Intergroup differences were assessed using two-sample *t*-tests with Gaussian random field correction.

**Results:**

VBM analysis revealed significant GMV reductions in the bilateral cerebellar crus I, left lobules I–IV, left crus II, left lobule IX, and right lobule VIIb in T2DM participants. Seed-to-voxel SCN analysis further demonstrated decreased covariance between the left crus I and the left middle temporal gyrus, middle occipital gyrus, and angular gyrus, along with increased covariance between the left lobules I–IV and the right caudate nucleus. Correlation analysis revealed that GMV of the left crus I was positively associated with Clock Drawing Test scores, while GMV of the right crus I was positively correlated with Auditory Verbal Learning Test (AVLT) scores. In addition, GMV of the right lobule VIIb was positively associated with both AVLT and Grooved Pegboard Test (GPT) scores, and GMV of the left lobule IX was positively correlated with GPT scores. With respect to network integrity, reduced SCN connectivity between the left crus I and the default mode network (DMN) was negatively correlated with AVLT and the color word test performance, whereas enhanced SCN connectivity between the left lobules I–IV and the right caudate nucleus was negatively correlated with AVLT scores and was positively correlated with Trail Making Test-A performance.

**Conclusion:**

By integrating VBM and SCN approaches, this study demonstrated that cerebellar GMV atrophy and abnormal structural covariance in T2DM were closely associated with cognitive dysfunction. These findings highlight the role of disrupted cerebro-cerebellar connectivity in the pathophysiology of T2DM-related cognitive impairment.

## Introduction

Diabetes mellitus (DM), a chronic metabolic disorder, has shown a progressive global increase in prevalence over recent decades (1). Epidemiological data from 2021 indicate that 537 million individuals worldwide were affected, of which 90–95% had type 2 DM (T2DM) (2, 3). Beyond its systemic complications, T2DM is strongly associated with central nervous system comorbidities, most notably mild cognitive impairment and dementia (4–6). Cognitive decline in T2DM typically affects multiple domains, including visuospatial processing, memory, attention, language, and executive functioning (7). Despite extensive research, the neuropathophysiological mechanisms underlying T2DM-related cognitive impairment remain incompletely understood.

The majority of prior investigations have focused on cerebral alterations, while cerebellar involvement has received comparatively limited attention. However, emerging evidence indicates cerebellar gray matter atrophy in T2DM (8–10). Cross-sectional and longitudinal neuroimaging studies further suggest that cerebellar gray matter volume (GMV) reduction and disrupted cerebro-cerebellar morphological network integrity may precede combined cognitive and motor decline in elderly populations (11).

Traditionally regarded as the primary center for motor coordination, the cerebellum is now recognized as a critical hub for higher-order cognitive processes (12–14). It contributes to attention, perception, memory, problem-solving, and decision-making (15). Through extensive reciprocal connections with cerebral cortical regions, the cerebellum plays an essential role in optimizing both motor and cognitive functions (16). Moreover, as a central node in large-scale functional networks, the cerebellum integrates multimodal sensory, motor, cognitive, emotional, and social information (8, 17, 18). These findings suggest that structural alterations in cerebellar gray matter and abnormal cerebro-cerebellar connectivity may significantly contribute to cognitive dysfunction in T2DM.

Despite accumulating evidence of cerebellar involvement in cognitive processes, current neuroimaging studies in T2DM have largely prioritized cerebral cortical and subcortical alterations, often neglecting the cerebellum. Most existing investigations reporting cerebellar changes in T2DM relied on whole-brain VBM approaches, which may lack the anatomical precision required to delineate subtle, region-specific cerebellar abnormalities. Furthermore, very few studies have systematically examined how structural alterations in discrete cerebellar subregions interact with cerebral networks to influence cognitive outcomes in T2DM. These gaps limit a comprehensive understanding of the cerebellum’s contribution to diabetes-related cognitive decline and underscore the need for regionally refined and connectivity-oriented analyses.

In this study, voxel-based morphometry (VBM) with the spatially unbiased infratentorial template (SUIT) was applied for precise subregional segmentation of the cerebellum, and regions exhibiting GMV alterations in T2DM were subsequently defined as regions of interest (ROIs) for seed-to-voxel structural covariance network (SCN) analyses. This dual approach was designed to: (1) characterize T2DM-related cerebellar GMV changes; (2) examine associated alterations in cerebro-cerebellar connectivity; and (3) evaluate their relationships with cognitive performance. By adopting a cerebellar-focused framework, this study aimed to provide novel insights into the neuropathophysiological mechanisms of cognitive impairment in T2DM.

## Methods

### Participants

From March 2024 to January 2025, we recruited 130 participants from the Endocrinology Department and Physical Examination Center of the First Affiliated Hospital of Guangzhou University of Chinese Medicine, comprising 73 cases with T2DM and 57 healthy controls (HCs). The inclusion criteria for all participants were: (1) aged 35–70 years; (2) right-handedness; and (3) ≥6 years of formal education. The exclusion criteria included: (1) organic central nervous system disorders (e.g., brain injury and tumors); (2) Parkinson’s disease, hyperthyroidism, or epilepsy; (3) history of alcoholism, substance abuse, or psychiatric disorders; (4) Fazekas scale score of 2 or 3; and (5) contraindication for magnetic resonance imaging (MRI). T2DM cases met the 2019 diagnostic criteria for diabetes with disease duration >1 year (World Health Organization). We excluded individuals with Montreal Cognitive Assessment (MoCA) scores ≤10, diabetic ketoacidosis, stage 4 diabetic nephropathy, diabetic foot, or other severe complications. All MRI scans were visually inspected by two experienced radiologists for motion artifacts and overall image quality. No participants were excluded due to excessive motion or poor image quality, resulting in a final cohort of 73 T2DM cases and 57 HCs. The study was conducted in accordance with the Declaration of Helsinki and approved by the Ethics Committee of the First Affiliated Hospital of Guangzhou University of Chinese Medicine. Written informed consent was obtained from all participants.

### Neuropsychological assessments

All participants completed the following standardized tests: Clock Drawing Test (CDT), Montreal Cognitive Assessment (MoCA), Digit Symbol Substitution Test (DSST), Auditory Verbal Learning Test (AVLT), Mini-Mental State Examination (MMSE), Digit Span Test (DST), Stroop Color-Word Test (CWT), Grooved Pegboard Test (GPT), and Trail Making Test Part A (TMT-A).

The MMSE and MoCA evaluated global cognitive function. The AVLT assessed memory, and the DSST measured visuomotor coordination. The TMT-A assessed processing speed and attention, and the DST evaluated working memory and attention. The GPT measured motor coordination and cognitive flexibility, the CDT assessed visuospatial abilities and executive function, and the CWT evaluated executive function and selective attention.

Raw scores from all neuropsychological tests were used in the analyses. To minimize the influence of demographic variability, age, sex, and years of education were included as covariates in subsequent statistical models rather than applying test-specific normalized scores. To minimize fatigue effects and potential order-related bias, all neuropsychological tests were administered in a fixed sequence, with brief rest periods provided between tests. The sequence was designed to progress from less demanding tasks (e.g., MMSE and MoCA) to more cognitively demanding or time-sensitive tasks (e.g., TMT-A, DSST, and CWT), ensuring consistency across participants. All cognitive tests were administered within 1 week of the MRI scan to minimize variability related to timing. The neuropsychological evaluations were conducted by trained examiners who were blinded to participants’ group allocation (T2DM or HC) to reduce potential assessment bias.

### Data acquisition

Neuroimaging was performed using a Siemens 3.0 T Prisma MRI scanner with a 64-channel head coil. Initial structural scans (T1-weighted, T2-weighted, and T2-FLAIR sequences) excluded participants with brain abnormalities. High-resolution T1-weighted images were acquired using sagittal 3D T1-weighted magnetization-prepared rapid gradient-echo (MPRAGE) sequences with the following parameters: echo time (TE) = 2.98 ms, repetition time (TR) = 2,530 ms, slice thickness = 1.0 mm, 192 slices; field of view (FOV) = 256 mm × 256 mm, flip angle = 7°, isotropic voxel size = 1 mm × 1 mm × 1 mm, and total acquisition time = 5 min 58 s.

### Structural MRI data processing

All software procedures were carried out in the MATLAB computational environment (version R2022b; MathWorks, Natick, MA, United States). Three-dimensional T1-weighted images were processed using the SUIT toolbox^1^ implemented within the Statistical Parametric Mapping software package (SPM12; https://www.fil.ion.ucl.ac.uk/spm). The preprocessing pipeline comprised the following sequential steps: (1) format conversion, in which DICOM image data were converted to NIfTI format; (2) spatial normalization, with anatomical origin aligned with the anterior commissure-posterior commissure (AC-PC) line, orientation verified, and resolution corrected; (3) tissue segmentation, including cerebellar isolation and gray matter segmentation; (4) spatial transformation, with normalization to SUIT standard space using the DARTEL algorithm; (5) atlas registration, achieved by reslicing into the SUIT cerebellar atlas space; and (6) spatial smoothing, with application of a 3-mm full width at half-maximum (FWHM) Gaussian kernel. Statistical analysis was performed using the DPABI 3.0 toolbox.^2^ Specifically, the VBM module was applied to examine group-level differences in regional GMV, and the seed-based SCN analysis module was used to assess covariance patterns of cerebellar subregions. In addition to cerebellar-focused preprocessing, whole-brain VBM analyses were performed using the Computational Anatomy Toolbox (CAT12) in SPM12 to explore potential regional cerebral gray matter alterations. The same preprocessing pipeline (segmentation, normalization to MNI space, modulation, and smoothing with an 8-mm FWHM Gaussian kernel) was applied for cerebral GMV estimation. In this study, a 3-mm FWHM Gaussian smoothing kernel was applied during structural MRI preprocessing using the SUIT toolbox. This choice follows recommendations from Diedrichsen (19) and Diedrichsen et al. (20), who highlighted that a 3-mm kernel preserves anatomical precision in cerebellar analyses. Specifically, Diedrichsen (19) noted that smoothing the final probability map using a 3-mm Gaussian kernel produces smooth segmentation edges. Moreover, the SUIT toolbox, developed by Diedrichsen and colleagues, facilitates accurate normalization of cerebellar structures into atlas space using the DARTEL algorithm and has been widely used with a 3-mm smoothing kernel in prior neuroimaging studies. All MR images were visually inspected by two experienced radiologists for motion artifacts, image distortions, and other anomalies prior to inclusion in analyses. Participants whose scans exhibited significant artifacts or failed quality control checks were excluded from further processing.

Between-group differences in GMV between T2DM participants and HCs were evaluated using two-sample *t*-tests. Cerebellar subregions demonstrating statistically significant GMV alterations were then defined as regions of interest (ROIs) for subsequent seed-to-voxel SCN analyses. To account for interindividual neuroanatomical variability, global tissue volume and total intracranial volume (TIV) were estimated in native T1 space using the *estimate TIV and global tissue volume* module within the Computational Anatomy Toolbox (CAT12).

### Statistical analysis

#### Demographic and clinical data analysis

Demographic and clinical variables were analyzed using SPSS 29.0 software (IBM Corp., Armonk, NY, United States). Continuous variables with normal distributions are presented as mean ± standard deviation (SD). Between-group differences were assessed using an independent samples *t*-test, while neuropsychological test scores were compared via analysis of covariance (ANCOVA). Categorical variables (sex and smoking status) were analyzed using the chi-squared test. A two-tailed *p*-value of <0.05 was considered statistically significant.

#### Identification of altered cerebellar subregions and structural covariance changes

Between-group differences in cerebellar gray matter structure were assessed using a two-sample *t*-test to identify significantly altered subregions. Group-level VBM comparisons of regional cerebral GMV between T2DM patients and HCs were also conducted. These analyses revealed no clusters of significant cerebral GMV reduction after correction for multiple comparisons, supporting a preferential vulnerability of cerebellar rather than cerebral structures in the current cohort. Six structurally distinct subregions were subsequently selected as seed ROIs for seed-to-voxel SCN analysis. For cerebral regions exhibiting structural covariance with the atrophic cerebellar ROIs, additional whole-brain VBM analyses were performed to determine whether these cortical and subcortical regions showed significant GMV reduction relative to controls. This step ensured that the observed structural covariance reflected coordinated morphological variation rather than secondary overlap with cerebral atrophy. The above statistical maps were corrected for multiple comparisons using Gaussian random field (GRF) theory (voxel level *p* < 0.001, cluster level *p* < 0.05), with years of education, TIV, sex, and age included as nuisance covariates.

#### Relationships between neuroanatomical metrics, clinical variables, and cognitive function

To examine the correlations between GMV in the identified cerebellar subregions, SCN integrity, clinical variables, and cognitive performance, partial correlation analyses were performed. Blood pressure, years of education, sex, smoking status, age, TIV, and disease duration were included as nuisance covariates. SCN integrity for each seed region was quantified by extracting the mean *z*-values of all voxels in significant clusters identified in the seed-to-voxel structural covariance analysis. These mean *z*-values were then utilized in partial correlation analyses to examine relationships with clinical variables and cognitive performance.

## Results

### Participants’ characteristics

Tables 1, 2 present the demographic, clinical, and neuropsychological characteristics of the two groups. No significant differences were observed between T2DM participants and HCs with respect to age, body mass index (BMI), sex distribution, years of education, low-density lipoprotein (LDL) levels, systolic blood pressure, smoking status, or total cholesterol levels (all *p* > 0.05). In contrast, the T2DM group showed significantly lower high-density lipoprotein (HDL) levels and higher fasting blood glucose, glycated hemoglobin (HbA1c), and triglyceride levels compared to HCs. Regarding cognitive performance, T2DM participants demonstrated significantly poorer outcomes on the MMSE, MoCA, AVLT, GPT, DSST, TMT-A, DST, and CWT (all *p* < 0.05), whereas no significant group difference was observed in CDT scores.

**Table 1:** Demographic and clinical features of the cases.

Variables	T2DM group (*n* = 73)	HC group (*n* = 57)	*p*-value
Age (years)	57.05 ± 4.91	55.82 ± 5.59	0.185^a^
Sex (men/women)	37 (36)	26 (31)	0.566^b^
Years of education (years)	9.09 ± 3.03	10.14 ± 2.98	0.052^a^
BMI (kg/m^2^)	23.65 ± 2.93	23.72 ± 2.99	0.839^a^
Systolic BP (mmHg)	125.00 ± 16.50	121.35 ± 9.22	0.079^a^
Diastolic BP (mmHg)	75.00 ± 8.50	78.61 ± 8.65	0.042^a^
Current smoker (never)	20 (53)	8 (49)	0.066^b^
Disease duration (years)	10.32 ± 6.60	—	
FPG (mmol/L)	7.54 ± 3.11	4.94 ± 0.39	<0.001^a^
HbA1c (%)	8.82 ± 4.03	5.49 ± 0.27	<0.001^a^
LDL (mmol/L)	3.22 ± 1.02	3.23 ± 0.77	0.681^a^
HDL (mmol/L)	1.13 ± 0.28	1.43 ± 0.43	<0.001^a^
TG (mmol/L)	1.87 ± 1.31	1.67 ± 1.83	0.040^a^
TC (mmol/L)	4.89 ± 1.54	5.09 ± 0.87	0.347^a^

All data are described as the mean ± standard deviation unless otherwise indicated.

BMI, body mass index; BP, blood pressure; FPG, fasting plasma glucose; LDL, low-density lipoprotein; HDL, high-density lipoprotein; TG, triglyceride; TC, total cholesterol.

a Two-sample *t*-test.

b Two-group chi-squared test.

**Table 2:** Neuropsychological test scores.

Variables	T2DM group (*n* = 73)	HC group (*n* = 57)	*p*-value
MoCA	24.85 ± 3.07	28.00 ± 1.22	<0.001^a^
MMSE	27.00 ± 1.80	29.00 ± 1.08	<0.001^a^
AVLT-3	8.49 ± 2.08	10.21 ± 1.76	<0.001^a^
AVLT-20 min	6.89 ± 2.39	9.61 ± 1.78	<0.001^a^
AVLT-R	10.52 ± 1.65	11.54 ± 0.82	<0.001^a^
GPT-R	89.33 ± 28.30	67.47 ± 12.19	<0.001^a^
GPT-L	96.56 ± 31.35	75.04 ± 19.25	<0.001^a^
DSST	29.7 ± 10.43	47.44 ± 14.92	<0.001^a^
CDT	3.49 ± 0.784	3.63 ± 0.67	0.335^a^
TMT-A	54.41 ± 20.13	39.44 ± 11.31	<0.001^a^
DST-FS	6.41 ± 1.30	7.98 ± 1.24	<0.001^a^
DST-BS	3.75 ± 1.09	4.79 ± 1.13	<0.001^a^
CWT(A)-RT	32.16 ± 10.94	26.61 ± 6.87	0.001^a^
CWT(B)-RT	47.58 ± 15.04	39.16 ± 14.04	<0.001^a^
CWT(C)-RT	87.52 ± 25.16	71.65 ± 21.98	<0.001^a^

All data are described as the mean ± standard deviation.

Re, recognition; R, right; L, left; FS, forward span; BS, backward span; RT, reading time.

a Two-group analysis of covariance adjusted for years of education, sex, and age.

### Cerebellar regions exhibiting altered GMV and structural covariance between groups

**Figure 1:**
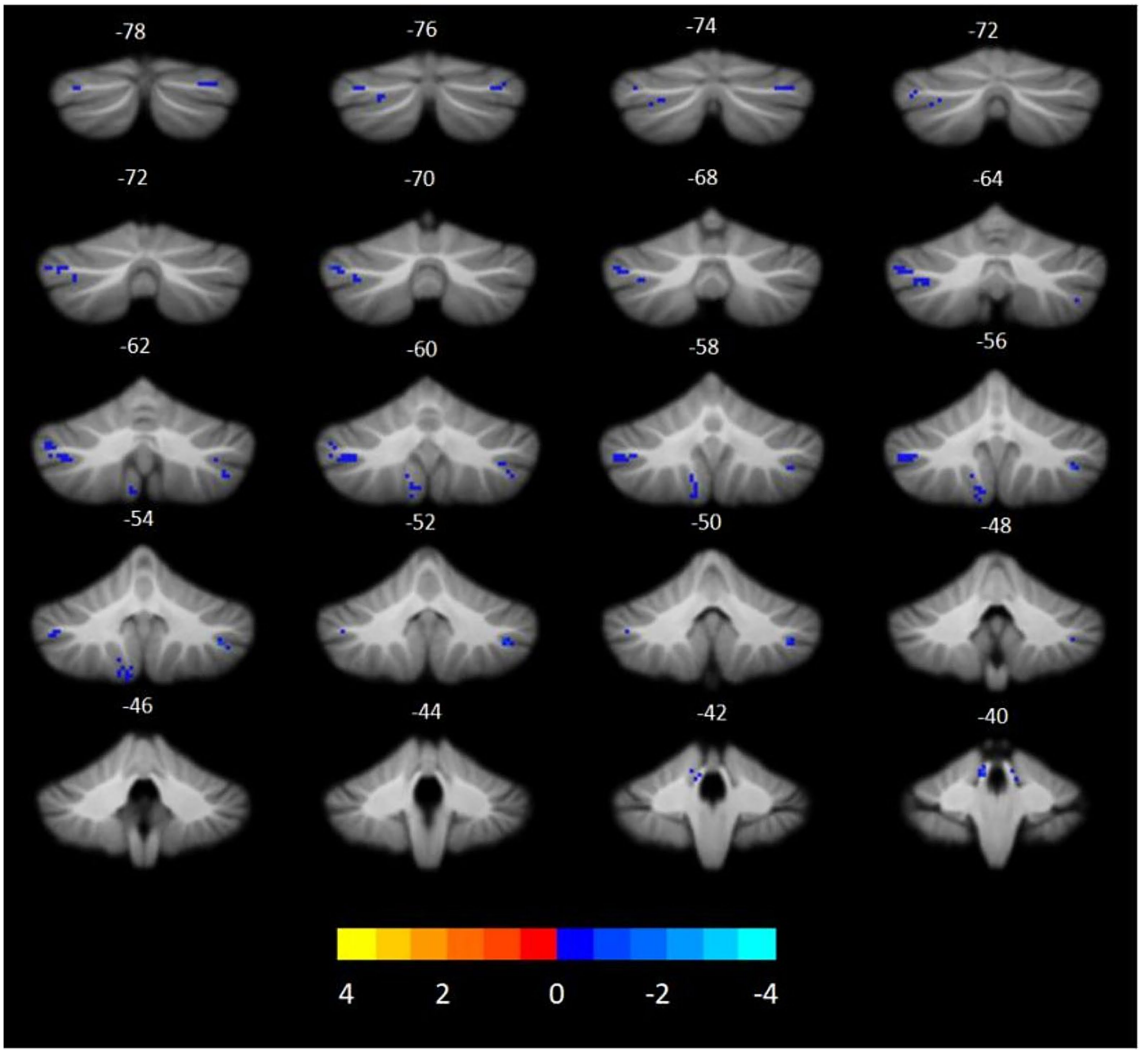
Cerebellar subregions with reduced GMV in T2DM compared to HCs. (GRF, voxel level *p* < 0.001, cluster *p* < 0.05, cluster size >19, coordinates are cerebellar coronal plane coordinates).

**Figure 2:**
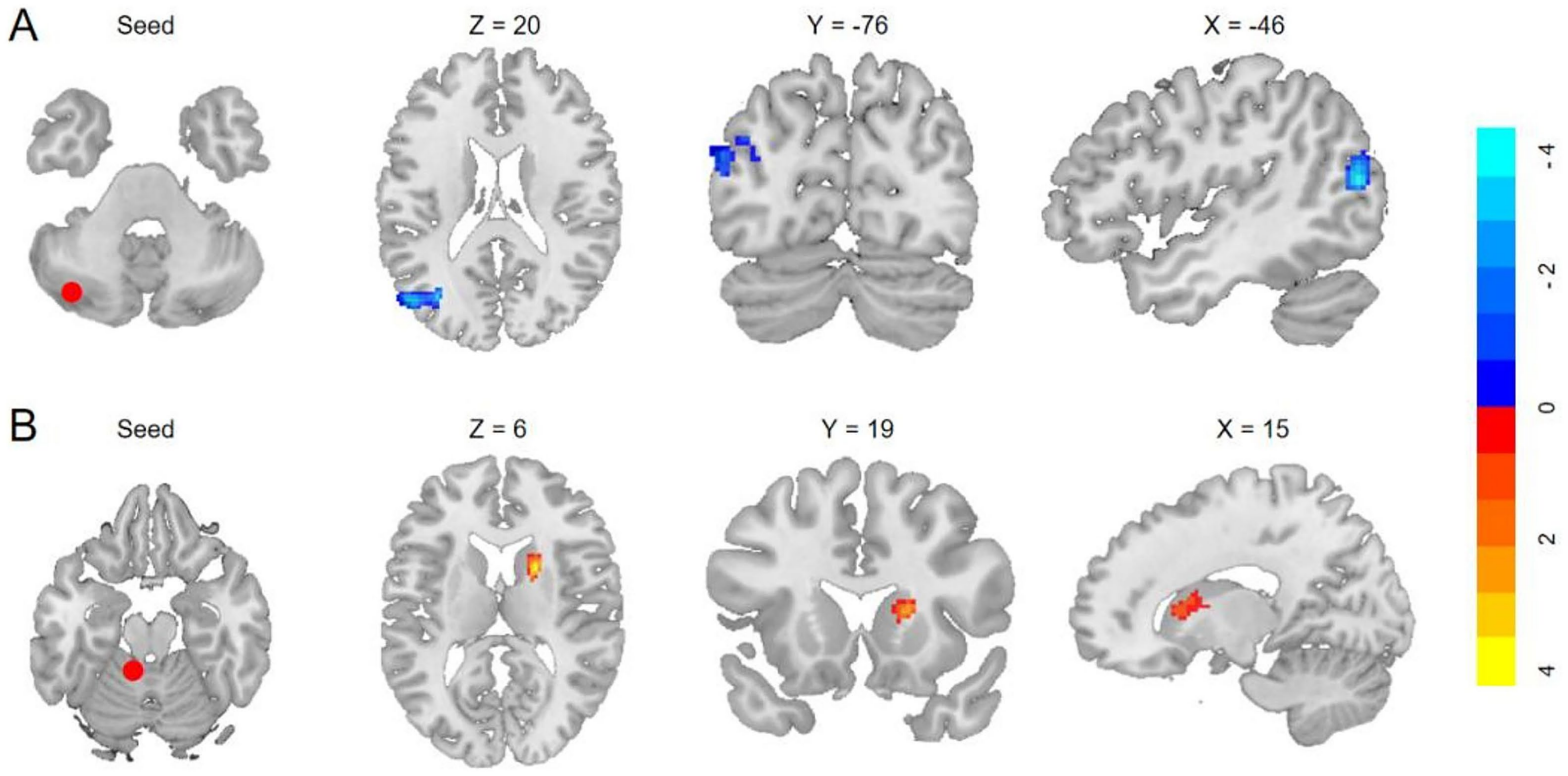
Anatomical regions with significantly altered structural covariance in T2DM. **(A)** Decreased structural covariances of the left crus I with left MTG/MOG/AG (GRF, voxelwise *p* < 0.001, cluster *p* < 0.05; cluster size >254 voxels). **(B)** Enhanced morphological covariances of the left I–IV with the right caudate (GRF, cluster *p* < 0.05, voxelwise *p* < 0.001; cluster size >196 voxels).

The VBM analysis revealed that, compared to HCs, participants with T2DM exhibited significant GMV reductions in the bilateral cerebellar crus I, left crus II, left lobules I–IV, left lobule IX, and right lobule VIIb. No cerebellar subregions demonstrated increased GMV in the T2DM group (Table 3 and Figure 1). Seed-to-voxel SCN analyses were then performed using these six GMV-reduced regions as ROIs. This analysis identified two cerebellar-based structural networks showing significant between-group differences. Specifically, reduced morphological covariance was observed between the left crus I and the left middle temporal gyrus, middle occipital gyrus, and angular gyrus, whereas increased morphological covariance was detected between the left lobules I–IV and the right caudate nucleus (Table 3 and Figure 2). Notably, none of the cerebral regions that covaried with cerebellar atrophy (e.g., middle temporal gyrus, angular gyrus, middle occipital gyrus, and caudate nucleus) exhibited significant GMV reduction in the between-group whole-brain VBM analysis, indicating that these areas were structurally preserved at the gross morphological level.

**Table 3:** Anatomical regions with altered structural covariance and cerebellar GMV in T2DM cases.

Maximum *t*-value	Cluster size	MNI coordinate (*x*, *y*, *z*)	Anatomical region
GMV (cluster size >19)			
−3.16248	63	−36, −68, −37	Left crus I
−3.16671	60	−14, −84, −27	Left crus II
−3.16396	21	32, −82, −29	Right crus I
−3.16195	32	−6, −54, −57	Left IX
−3.19313	28	38, −58, −45	Right VIIb
−3.33153	25	−8, −39, −20	Left I–IV
Structural covariance (seed: left crus I, T2DM <HCs; cluster size >254)			
−3.15615	460	−56, −70, −20	Left MTG/MOG/AG
Structural covariance (seed: left I–IV, T2DM >HCs; cluster size >196)			
3.85616	206	21,22,6	Right caudate

GMV, gray matter volume; MTG, middle temporal gyrus; MOG, middle occipital gyrus; AG, angular gyrus; MNI, Montreal Neurological Institute and Hospital. Gaussian random field correction (GRF, cluster *p* < 0.05, voxel *p* < 0.001).

### Correlation analysis

#### Relationship between reduced cerebellar GMV and clinical characteristics in T2DM cases

**Figure 3:**
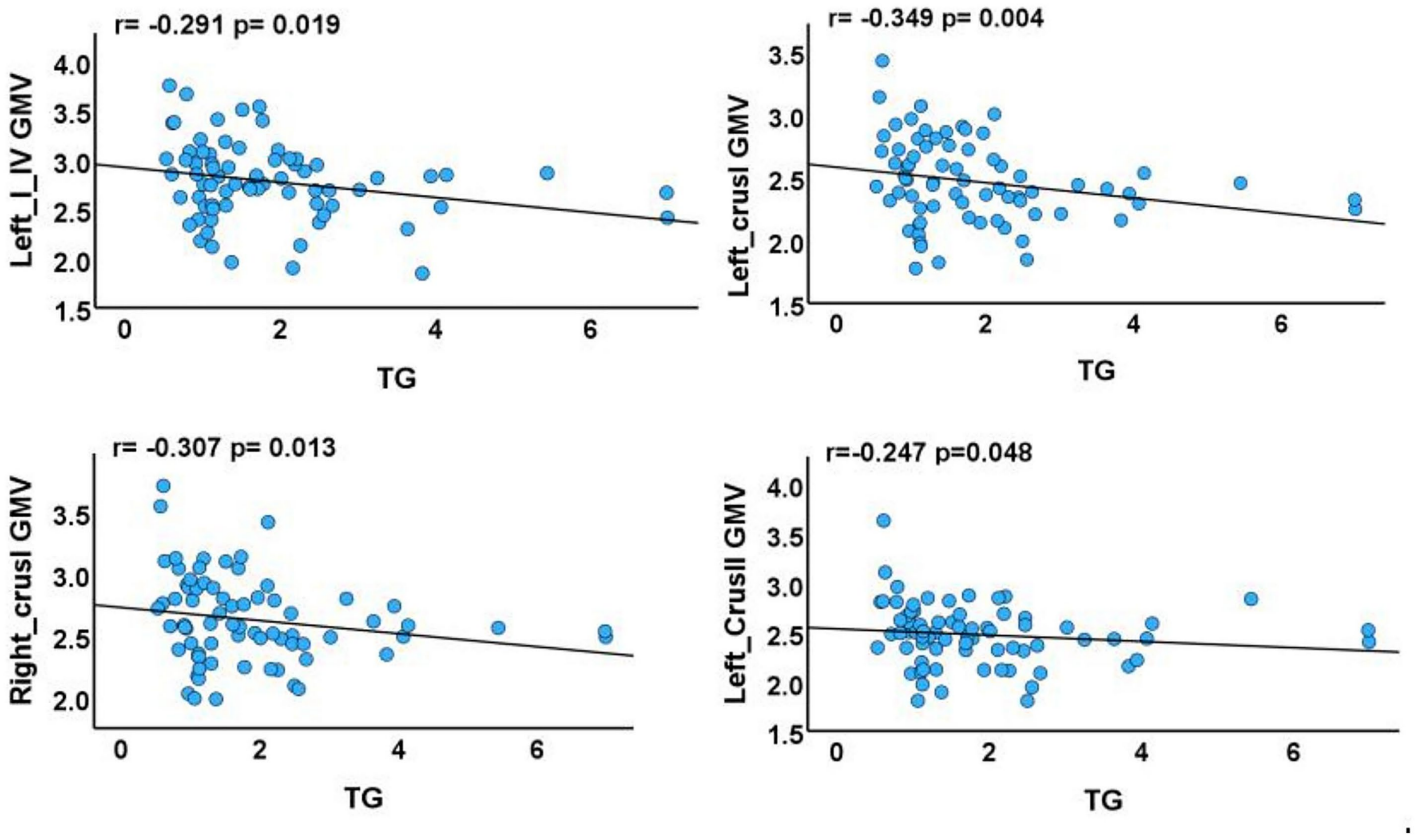
Associations between cerebellar subregions with reduced GMV and clinical characteristics of T2DM cases. GMV, gray matter volume; TG, triglyceride. The results of partial correlation analyses after adjustment for smoking status, sex, age, disease duration, blood pressure, years of education, and total intracranial volume (*p* < 0.05).

The results demonstrated negative correlations between GMV in the left crus I, left crus II, right crus I, left lobules I–IV, and triglyceride levels (*p* = 0.004, *r* = −0.349; *p* = 0.048, *r* = −0.247; *p* = 0.013, *r* = −0.307; *p* = 0.019, *r* = −0.291, respectively) (Figure 3). No significant correlations were observed between GMV in the right lobule VIIb or left lobule IX and clinical characteristics.

#### Relationship between reduced cerebellar GMV and cognitive function in T2DM cases

**Figure 4:**
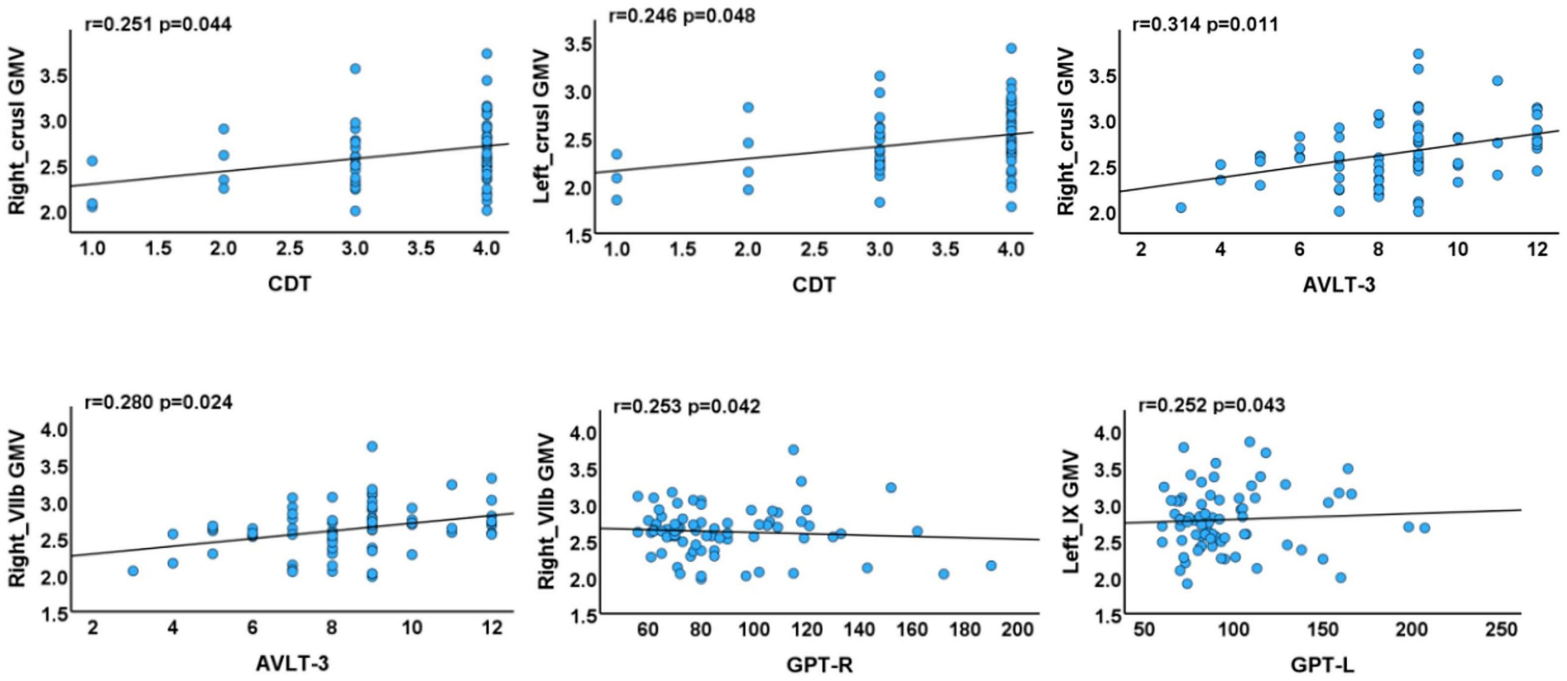
Associations between cerebellar subregions with reduced GMV and neuropsychological test scores of T2DM cases. AVLT, Auditory Verbal Learning Test; GPT, Grooved Pegboard Test; CDT, Clock Drawing Test (R, right, L, left).

Positive correlations were identified between: (1) GMV in the left crus I and CDT scores (*p* = 0.048, *r* = 0.246); (2) GMV in the right crus I and AVLT-3 scores (*p* = 0.011, *r* = 0.314); (3) GMV in the right lobule VIIb and both AVLT-3 and GPT-R scores (*p* = 0.024, *r* = 0.280; *p* = 0.042, *r* = 0.253); and (4) GMV in the left lobule IX and GPT-L scores (*p* = 0.043, *r* = 0.252) (Figure 4).

#### Relationship between seed-based SCN integrity and cognitive function

**Figure 5:**
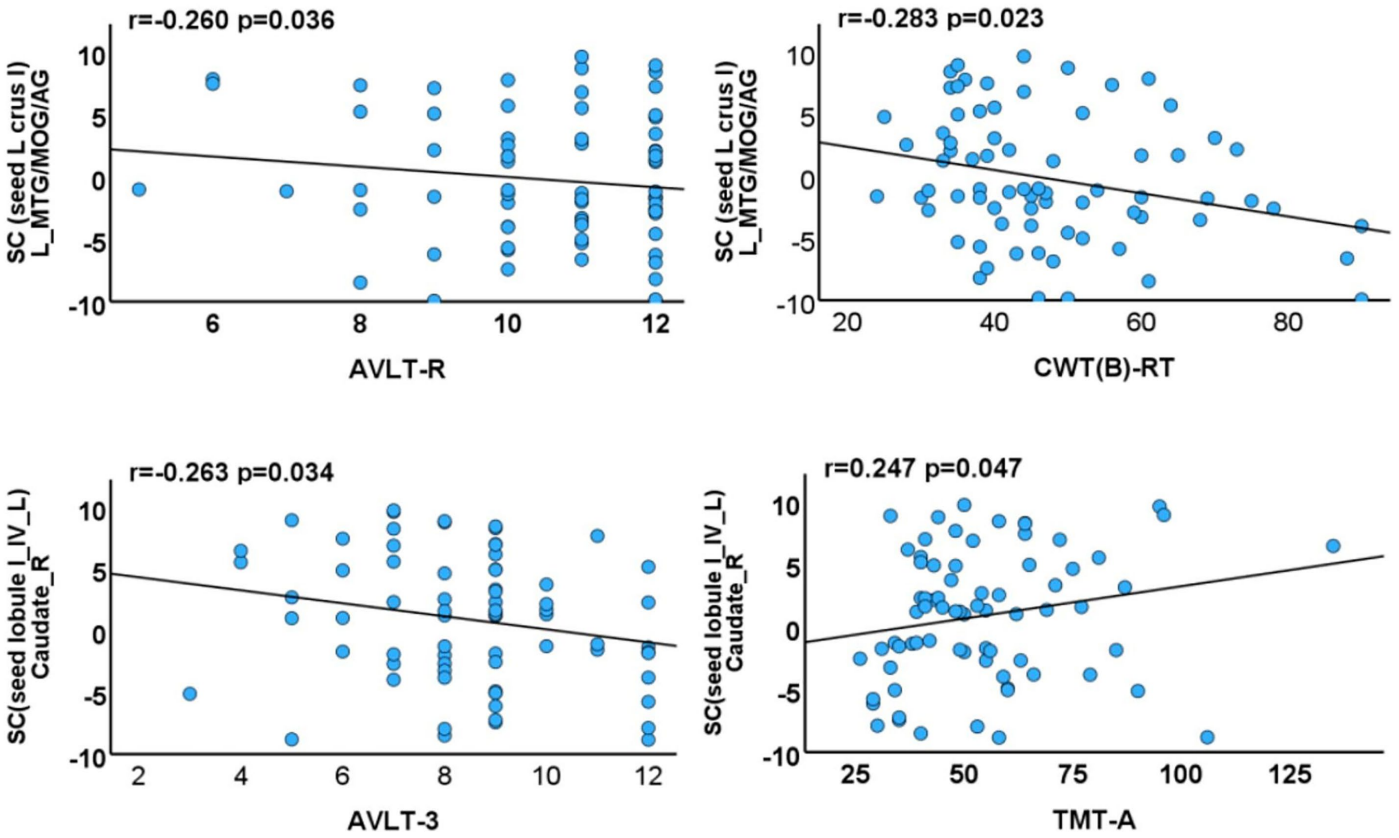
Associations between cerebellar subregions with reduced GMV/morphological network integrity and neuropsychological test scores of T2DM cases. SC, structural covariance; TIV, total intracranial volume; MTG, middle temporal gyrus; MOG, middle occipital gyrus; AG, angular gyrus; AVLT-R, Auditory Verbal Learning Test-Recognition; CWT(B)-RT, Stroop Color-Word Test Part B-Reading Time; TMT-A, Trail Making Test Part A.

Negative correlations were identified between SCN integrity and cognitive performance. Specifically, reduced integrity between the left crus I and the default mode and visual networks (left middle temporal gyrus, angular gyrus, and middle occipital gyrus) was associated with poorer long-term memory (Auditory Verbal Learning Test-Recognition, AVLT-R; *p* = 0.036, *r* = −0.260) and executive function (CWT B-RT; *p* = 0.023, *r* = −0.283). In addition, decreased integrity between the left lobules I–IV and the right caudate nucleus was correlated with lower short-term memory performance (AVLT-3; *p* = 0.034, *r* = −0.263). Conversely, increased integrity between the left lobules I–IV and the right caudate nucleus was positively correlated with processing speed/attention (TMT-A; *p* = 0.047, *r* = 0.247) (Figure 5).

To reduce potential confounding influences of clinical variables and to isolate neuroanatomical correlates of cognitive dysfunction in T2DM, all analyses were adjusted for age, sex, years of education, TIV, blood pressure, smoking status, and disease duration. A threshold of *p-*value of <0.05 was considered statistically significant.

## Discussion

This study elucidated the relationship between regional GMV alterations, SCN changes, and cognitive function in patients with T2DM. Compared to HCs, T2DM patients exhibited cerebellar GMV atrophy. Seed-to-voxel SCN analysis revealed reduced morphological network integrity between the left crus I and both the DMN and the VN, alongside enhanced morphological network integrity between the left lobules I–IV and the right caudate nucleus. Correlation analyses demonstrated significant associations between these GMV alterations/SCN measures and multiple cognitive domains, including executive function, memory, and processing speed/attention. In this study, covariates were selected based on their known associations with GMV, structural covariance, and cognitive function, including age, sex, duration of education, TIV, blood pressure, smoking status, and disease duration. Multicollinearity among covariates was evaluated using variance inflation factors (VIFs), and all VIFs were below 2, indicating no substantial collinearity that could bias the analyses. Although smoking status did not significantly differ between groups, it was included as a covariate because smoking is associated with cerebrovascular alterations, GMV reductions, and cognitive decline, which could confound neuroimaging findings independent of T2DM. Collectively, these findings provide a neuroanatomical basis for understanding T2DM-associated cognitive dysfunction.

### Cerebellar GMV atrophy and cognitive decline in T2DM cases

T2DM patients exhibited significant GMV reductions in the bilateral cerebellar crus I, left crus II, left lobules I–IV, left lobule IX, and right lobule VIIb. These findings largely align with previous reports of GMV reductions in the right crus I and bilateral lobules I–V/I–IV in T2DM (21). Notably, previous studies did not systematically assess the relationships between these cerebellar structural alterations and cognitive function.

The anterior lobe of the cerebellum plays a central role in regulating muscle tone, maintaining postural balance, and coordinating voluntary movements (22, 23). Atrophy within this region in T2DM may therefore contribute to impaired motor coordination and reduced fine motor control. Consistent with this, the present study demonstrated that participants with T2DM exhibited significantly poorer motor coordination and diminished neurocognitive flexibility compared to HCs.

Beyond its motor functions, the cerebellum also contributes to higher-order cognition, particularly through the posterolateral hemisphere. Cognitive tasks typically engage three functionally distinct, non-motor cerebellar regions: (1) lobule VI–crus I, (2) crus II–VIIB, and (3) lobules IX–X (24–26). Our neuroimaging results showed that GMV reductions in these posterolateral regions were significantly associated with multidomain cognitive impairments, including visuospatial deficits, executive dysfunction, impaired memory consolidation, disrupted limb coordination, and reduced neurocognitive flexibility. Notably, bilateral crus I/II are key nodes within networks supporting executive function, working and episodic memory, navigation, and attentional control (27–30), while lobules VI and VII are typically engaged during reading tasks (31). These converging findings confirm the present results and indicate that cerebellar degeneration constitutes a critical neuroanatomical substrate underlying T2DM-related cognitive impairment.

### Reduced SC between the left crus I and DMN

Notably, structural covariance (SC) is understood to reflect both structural and functional connectivity (32), and cerebro-cerebellar covariance patterns are thought to mirror the underlying anatomical coupling between sensorimotor and cognitive networks (33). In the present study, SCN analysis revealed decreased connectivity between the left crus I and both the DMN and the VN. Importantly, reduced SCN integrity in these pathways was negatively correlated with memory and executive performance in T2DM, suggesting that disrupted cerebro-cerebellar structural connectivity constitutes a potential neural mechanism underlying cognitive impairment.

Crus I/II has been identified as the principal cerebellar hub interfacing with the DMN (24, 29). Consistent with our findings, previous resting-state fMRI studies have reported disrupted and reorganized functional connectivity between the cerebellum and the DMN in T2DM (34). The present results, therefore, not only highlight the role of the cerebellum as a critical neuroanatomical substrate for T2DM-related cognitive decline but also emphasize that aberrant morphological connectivity within cerebro-cerebellar DMN/VN circuits contributes to the pathophysiology of this dysfunction. Taken together, converging evidence from structural and functional network studies strengthens the reliability of these findings. It may be hypothesized that the left crus I represents a neuroanatomically vulnerable locus, or an early-affected region, mediating cognitive decline in T2DM.

### Enhanced SC between the cerebellum and caudate nucleus

The caudate nucleus is critically involved in both motor and cognitive domains, contributing to motor planning/execution, spatial working memory, reward processing, motivation, and emotion (35–37). Aberrations in caudate networks have been recognized as predictors of cognitive decline in aging and neurodegenerative conditions (38, 39). Notably, the present study revealed enhanced structural connectivity between the left lobules I–IV and the right caudate nucleus. This connectivity was negatively correlated with short-term memory performance (AVLT-3), while it was positively correlated with processing speed and attention (TMT-A).

Previous studies have reported increased caudate-cortical connectivity in elderly populations exhibiting dual cognitive and motor decline (11). This finding may thus represent a unique neuroanatomical feature of T2DM-related cognitive dysfunction. Whether this connectivity enhancement reflects a maladaptive change or a compensatory mechanism remains uncertain and warrants further mechanistic investigation.

### Limitations

Several methodological limitations warrant consideration: First, the cross-sectional design with a relatively small sample size limits causal inference. Second, individual variability in hypoglycemic medications precluded assessment of their effects on cerebellar subregional GMV. Third, the potential confounding effects of mild microvascular complications in some participants remain unaddressed. Future studies should examine cerebellar structural changes across different T2DM progression stages to better characterize neuroimaging biomarkers of cerebellar impairment.

## Conclusion

This study demonstrated significant associations between T2DM-related cognitive dysfunction and cerebellar GMV atrophy. Seed-to-voxel SCN analysis revealed both impaired connectivity with the default mode and visual networks and enhanced connectivity with the caudate nucleus. These findings highlight the critical role of cerebellar degeneration in T2DM-associated cognitive impairment and further indicate that abnormal structural connectivity in cerebro-cerebellar circuits contributes to its pathophysiology. Collectively, the results provide novel insights into the neuropathophysiological mechanisms underlying cognitive decline in T2DM.

## Data Availability

The original contributions presented in the study are included in the article/supplementary material, further inquiries can be directed to the corresponding author.

## Footnotes

^1^ https://www.diedrichsenlab.org/imaging/suit_function.htm

^2^ http://rfmri.org/dpabi
